# Evaluation of sensitivity and specificity of the INECO Frontal Screening and the Frontal Assessment Battery in mild cognitive impairment

**DOI:** 10.1590/1980-57642021dn15-010010

**Published:** 2021

**Authors:** Zoylen Fernández-Fleites, Elizabeth Jiménez-Puig, Yunier Broche-Pérez, Sheyla Morales-Ortiz, Darlyn Alejandra Reyes Luzardo, Luis Ramón Crespo-Rodríguez

**Affiliations:** 1 Psychology Department, Universidad Central “Marta Abreu” de Las Villas – Santa Clara, Cuba.; 2 CognitiON (Cuban Inicative on Cognitive Health) – Santa Clara, Cuba.; 3 Universidad Laica “Eloy Alfaro” de Manabí – Manta, Ecuador.; 4 Deparment of Neurology, Arnaldo Milián Castro Provincial Hospital – Santa Clara, Cuba.

**Keywords:** mild cognitive impairment, cognitive assessment screening instrument, sensitivity, specificity, disfunção cognitiva, testes de estado mental e demência, sensibilidade, especificidade

## Abstract

The Frontal Assessment Battery (FAB) and the INECO Frontal Screening (IFS) are two instruments frequently used to explore cognitive deficits in different diseases. However, studies reporting their use in patients with mild cognitive impairment (MCI) are limited.

**Objective:**

To compare the sensitivity and specificity of FAB and IFS in mild cognitive impairment (multiple-domain amnestic MCI subtype — md-aMCI).

**Methods:**

IFS and FAB were administered to 30 md-aMCI patients and 59 healthy participants. Sensitivity and specificity were investigated using the Receiver Operating Characteristic (ROC) analysis.

**Results:**

The area under the ROC curve (AUC) of IFS for MCI patients was .82 (sensitivity=0.96; specificity=0.76), whereas the AUC of FAB was 0.74 (sensitivity=0.73; specificity=0.70).

**Conclusions:**

In comparison to FAB, IFS showed higher sensitivity and specificity for the detection of executive dysfunctions in md-aMCI subtype. The use of IFS in everyday clinical practice would allow detecting the frontal dysfunctions in MCI patients with greater precision, enabling the early intervention and impeding the transition to more severe cognitive alterations.

## RESUMO.

A Bateria de Avaliação Frontal (FAB) e o teste de rastreio frontal do INECO (IFS) são dois instrumentos frequentemente utilizados para explorar déficits cognitivos em diferentes doenças. No entanto os estudos que relatam seu uso em pacientes com comprometimento cognitivo leve (MCI) são limitados.

**Objetivo:**

Comparar a sensibilidade e especificidade da FAB e IFS em comprometimento cognitivo leve (subtipo amnéstico de múltiplos domínios [md-aMCI]).

**Métodos:**

O IFS e FAB foram administrados a 30 pacientes md-aMCI e 59 participantes saudáveis. A sensibilidade e a especificidade foram exploradas usando a análise ROC.

**Resultados:**

A área sob a curva ROC (AUC) do IFS para pacientes com MCI foi de 0,82 (sensibilidade=0,96; especificidade=0,76), enquanto a AUC de FAB foi de 0,74 (sensibilidade=0,73; especificidade=0,70).

**Conclusões:**

Em comparação com o FAB, o IFS apresentou maior sensibilidade e especificidade para detecção de disfunções executivas no subtipo md-aMCI. O uso do INECO Frontal Screening (IFS) na prática clínica cotidiana, permitiria detectar com maior precisão as disfunções frontais em pacientes com deficiência cognitiva leve, possibilitando a intervenção precoce, impedindo a transição para alterações cognitivas mais graves.

## INTRODUCTION

Mild cognitive impairment (MCI) is considered a transition phase between normal cognition and Alzheimer's disease (AD).^1^ The prevalence of MCI in people aged over 65 years ranges from 7 to 47.9%, with a global prevalence of 18.9% per one thousand people.^2^ MCI patients can be classified in two main categories: amnestic MCI (aMCI), if patients show a poor performance on the episodic memory test, but functioning in other cognitive domains is preserved; and non-amnestic MCI (naMCI), if patients show a poor performance on cognitive evaluation covering domains other than memory such as language, visuospatial abilities, or executive functions.^2^ Additionally, aMCI patients could be classified in one of two possible clinical subtypes: (i) single-domain aMCI (sd-aMCI), when memory is the only impaired domain; and (ii) multiple-domain aMCI (md-aMCI), when besides the memory deficit, at least another cognitive domain is impaired (e.g., executive function, language, or visuospatial abilities).^3^

The annual conversion of MCI to AD is estimated between 10 and 15%.^4^ Approximately 50% of people with MCI will be diagnosed with AD in the following 4 years.^1^. However, the specific conversion prognoses for each subtype may differ. In this sense, md-aMCI patients had more severe deficit in working memory and problem solving than sd-aMCI patients, leading to the assumption that this subtype, not pure amnestic MCIs, are at highest risk of dementia.^5^ ^,^ ^6^ Another study sought to more systematically and comprehensively investigate predictors of rate of cognitive decline in a longitudinal sample of individuals with MCI, including age, genetic vulnerability, baseline cognitive performance, and baseline neuropsychiatric severity.^7^ The results showed that participants with composite scores for lower executive functions and greater severity of memory impairment at baseline predicted faster decline on dementia severity measures.

Recently, a meta-analytic study was conducted to explore inhibitory control (IC) in patients with aMCI, using a battery of well-validated inhibition tasks.^8^ According to the findings, patients with aMCI showed a generalized IC deficit, suggesting that inhibition paradigms should be routinely included in neuropsychological evaluations to obtain a more detailed overview of executive functioning in MCI patients.

Hence, early diagnosis of pathological cognitive decline becomes increasingly important in providing patients with the necessary interventions.^9^ Global neuropsychological batteries, such as the Montreal Cognitive Assessment (MoCA), the Mini-Mental State Examination (MMSE), and the Mattis Dementia Rating Scale second edition (DRS-2), are frequently used for neuropsychological evaluation. These screening batteries are helpful for obtaining a global cognitive performance approximation; however, they do not allow researchers to delve into executive functioning.

In this sense, the Frontal Assessment Battery (FAB) and the INECO Frontal Screening (IFS) are two frequently used instruments to explore frontal dysfunctions in different pathologies.^10^ FAB was designed for brief investigation of executive functioning and consists of six subtests that evaluate mental flexibility, conceptualization, inhibitory control, motor programming, resistance to interference, and environmental autonomy.^11^ This instrument is fundamentally used for two purposes:^12^

1. early identification of neurodegenerative diseases, and
2. detection of executive dysfunctions in different diseases that affect the frontostriatal brain circuits.

On the other hand, IFS is a brief neuropsychological test designed to explore executive functions across neurodegenerative pathologies such as AD,^13^ behavioral variant frontotemporal dementia,^14^ Multiple Sclerosis (relapsing-remitting phase),^15^ MCI,^16^ and MCI in Parkinson's disease.^17^ IFS is composed of eight subtests, organized into three main executive domains:^18^

1. inhibition and change,
2. working memory, and
3. capacity for abstraction.

Some studies have compared the clinical utility of FAB and IFS. For example, Gleichgerrcht et al.^19^ evaluated the usefulness of FAB and IFS in a group of 25 patients diagnosed with behavioral variant frontotemporal dementia (bvFTD) and 25 patients with AD. Compared with FAB, IFS showed a better capacity to discriminate between both dementia subtypes, a greater sensitivity and specificity for the detection of executive dysfunctions, and a high correlation with frequently used executive tests such as the Trail Making Test (part B) and the Wisconsin Card Sorting Test.

This result was confirmed by another study carried out on Peruvian patients.^20^ In this investigation, the diagnostic capacity of FAB and IFS was compared in a sample of 117 participants (35 AD patients, 34 patients with bvFTD, and 48 healthy controls [HC]). IFS showed a greater sensitivity to discriminate between AD and bvFTD, compared with FAB. The objective of the present study is to compare the sensitivity and specificity of FAB and IFS in patients with multiple-domain aMCI subtype (md-aMCI). Considering previous studies that have used both instruments, the authors’ hypothesis is oriented towards a better sensitivity of IFS compared with FAB.

## METHODS

### Participants

A total of 89 participants were evaluated: 59 cognitively healthy controls and 30 patients with MCI (multiple-domain aMCI subtype). The groups were selected according to the following criteria:

### Healthy control group

The following criteria were used to select the control group: scoring more than 85 points on the Addenbrooke's Cognitive Examination Revised (ACE-R),^21^ no subjective complaints of memory, and preserved functioning in the activities of daily living. Psychiatric history was reported by the participants during the initial interview (conducted by an experienced psychiatrist) and an experienced neurologist performed the neurological examination.

### Mild cognitive impairment Group (multiple-domain aMCI subtype)

MCI patients were classified using the criteria proposed by Petersen:^2^ objective impairment in formal neuropsychological measures (total score in ACE-R of at least 1.5 standard deviation [SD]below the demographically corrected mean)^21^ and preservation of activities of daily living (Barthel index>95).^22^ Patients with potential causes of cognitive deficits different than neurodegenerative or cerebrovascular disease (e.g., Schizophrenia, alcoholism, epilepsy, depression, head injury) were excluded. According to the standardized neuropsychological assessment, the sample was classified as multiple-domain aMCI subtype.

Patients with MCI who showed clinical signs of depression (Geriatric Depression Scale<5)^23^ or anxiety (Zung Self-Rating Depression Scale<51) were excluded from the study.^24^ The presence of severe sensory deficits (vision and hearing) was also considered an exclusion criterion.

### Instruments

#### Frontal Assessment Battery

The FAB^11^ is a test battery easy to administer and sensitive to frontal dysfunction. FAB consists of six subtests evaluating similarities (conceptualization), motor programming, inhibitory control, verbal fluency (mental flexibility), resistance to interference, and environmental autonomy. Each subtest is scored on a maximum of three points, with a total score of 18. High scores indicate preservation of the executive functions.

#### INECO Frontal Screening

INECO Frontal Screening (IFS)^14^ is a brief neuropsychological test battery to explore executive functioning in neurodegenerative diseases. The subtests included in IFS are Luria's Fist-Edge-Palm task (three points), Conﬂicting instructions (sensitivity to interference) (three points), Inhibitory control (three points), Months backwards (verbal working memory) (two points), Digit Span Task (six points), Corsi Block Tapping Test (four points), Proverb interpretation (abstraction capacity) (three points), and Verbal inhibitory control (modified Hayling Test) (six points). IFS has a maximum possible score of 30 points. High scores indicate preservation of the executive functions.^25^

#### Procedure and Analysis of Data

All participants were informed of the objectives of the study and signed the informed consent form. All cognitive evaluations were done blindly (HC vs MCI) and independently by one neuropsychologist who applied the IFS and FAB tests to all the participants. IFS and FAB were applied in different sessions to reduce fatigue and potential learning effects.

Data were obtained following the regulations of the ethics committee of the Department of Psychology of Universidad Central “Marta Abreu” de Las Villas and in accordance with the Declaration of Helsinki. Data were processed using the *Statistical Package for the Social Sciences* (SPSS) for Windows, version 21. Descriptive statistics were used to explore participants’ characteristics. An independent-sample Student's t-test was conducted to compare the executive functioning between groups. In all analyses, the homogeneity of variances was considered (by using the Levene's test for homogeneity of variances). The Cohen's *d* effect size was calculated to estimate effect sizes in all comparisons. Values above 0.2, 0.5, and 0.8 were considered as small, medium, and large effect size, respectively.^26^ Linear regression was used to evaluate age and education effects over total scores of FAB and IFS. To investigate the sensitivity and specificity of FAB and IFS, the Receiver Operating Characteristic (ROC) curve analysis was performed.

## RESULTS

### Demographics of mild cognitive impairment patients and Control Group

The present study was conducted with 59 healthy participants and 30 patients with MCI diagnosis (multiple-domain aMCI subtype). The results of the comparison of demographics are summarized in Table 1. There are no significant differences in age, education years, and sex between groups. Signiﬁcant differences were found between groups regarding the Addenbrooke's Cognitive Examination Revised (ACE-R).

**Table 1:** Demographic and neuropsychological characteristics of Healthy Controls and Mild Cognitive Impairment patients.

		HC (n=59)		MCI (n=30)		p-value
		M	SD	M	SD	
Age (years)		76.25	8.08	78.63	8.26	0.84
Education level (years)		12.38	4.15	11.63	3.07	0.63
Sex (M_a_:F)		29–30		15-15		0.94
Handedness (R:L)		59–0		29_1		0.15
ACE-R (/100)		89.74	4.36	68.13	11.01	<0.001
	Attention and Orientation (/18)	16.67	1.67	13.96	2.31	<0.001
	Memory (/26)	23.11	2.16	17.53	4.53	<0.001
	Verbal Fluency (/14)	10.06	1.89	5.90	1.82	<0.001
	Language (/26)	25.20	1.18	20.86	3.47	<0.001
	Visuospatial (/16)	14.61	1.48	10.03	2.89	<0.001

HC: healthy controls; MCI: mild cognitive impairment patients; M: mean; SD: standard deviation; M_a_: male; F: female; R: right; L: left; ACE-R: Addenbrooke's Cognitive Examination Revised.

Overall, age had no influence on FAB (r=-0.086, p=0.27) and IFS (r=-0.082, p=0.18), whereas years of education had a positive linear influence on FAB (r=0.32, p<0.001) and IFS (r=0.29, p=0.004).

### Subtests shared by both INECO Frontal Screening and Frontal Assessment Battery

FAB and IFS shared three subtests:^19^ Luria's Fist-Edge-Palm task, Conﬂicting instructions, and Inhibitory control (Go/No-go task) (Table 2). Two subtests showed differences between groups. Resistance to interference (Conflicting instructions) showed differences between groups. The MCI group showed lower scores than the HC group. The means of the scores in the Inhibitory control (Go/No-go task) also showed significant differences between groups. Patients with MCI performed worse than healthy controls. No differences between HC group and MCI patients were found in Luria's Fist-Edge-Palm task.

**Table 2:** Performance of healthy controls and mild cognitive impairment patients in the Frontal Assessment Battery.

FAB subtests	HC	MCI	t	p-value	d
	(n=59)	(n=30)			
	M(SD)	M(SD)			
Conceptualization	2.29(0.76)	1.83(0.74)	2.66	0.009	0.61
Mental flexibility	2.51(0.67)	1.97(0.71)	3.49	0.001	0.79
Motor programming*	2.42(0.77)	2.23(1.04)	0.97	0.33	0.22
Resistance to interference*	2.66(0.63)	2.26(0.86)	2.44	0.017	0.56
Inhibitory control*	2.10(0.90)	1.36(0.96)	3.54	0.001	0.81
Environmental autonomy	2.93(0.41)	2.87(0.43)	0.69	0.48	0.14
Total	14.85(2.33)	12.47(2.78)	4.27	<0.001	0.96

* Subtests shared by both IFS and FAB tests. IFS: INECO Frontal Screening; FAB: Frontal Assessment Battery; HC: healthy controls; MCI: mild cognitive impairment patients; SD: standard deviation; M: mean, d: effect size.

#### Frontal Assessment Battery

The results of HC participants and MCI patients concerning the FAB test is summarized in Table 2. In addition to the test shared by both instruments, the subtests Conceptualization, Mental flexibility, and Total score differed between groups. In all cases, MCI patients showed lower scores than HC participants. In Conceptualization and Mental Flexibility subtests, the effect size of the differences was medium (d>0.5). For Total Score in FAB, differences between means was large (d>0.8). No differences between MCI patients and the HC group were found in the Environmental Autonomy domain.

#### INECO Frontal Screening

The executive functioning of MCI patients and HC participants according to IFS is summarized in Table 3. In Backward months (Verbal working memory), Corsi Block Tapping Test (Spatial working memory), Modified Hayling Test (Verbal inhibitory control), and IFS total score showed significant differences between groups. In all cases, MCI patients showed poorer performance than HC participants. In verbal and spatial working memory domains, the effect size of the differences in the means was medium (d>0.5). For Verbal inhibitory control (Modified Hayling Test) and IFS total score, differences between means was large (d>0.8). No differences between MCI patients and the HC group group were found in the Working memory (Digit Span Task), and Abstraction capacity (Proverb interpretation) domains.

**Table 3:** Performance of healthy controls and mild cognitive impairment patients in the INECO Frontal Screening.

IFS subtests	HC	MCI	t	p-value	d
	(n=59)	(n=30)			
	M(SD)	M(SD)			
Motor series*	2.42(0.77)	2.23(1.04)	0.97	0.33	0.22
Conflicting instructions*	2.66(0.63)	2.26(0.86)	2.44	0.017	0.56
Go/No-go task*	2.10(0.90)	1.36(0.96)	3.54	0.001	0.81
Digit Span Task	2.67(1.04)	2.46(0.81)	0.96	0.33	0.21
Verbal working memory	1.69(0.50)	1.26(0.69)	3.34	0.001	0.76
Spatial working memory	2.20(0.97)	1.63(0.92)	2.64	0.01	0.60
Proverb interpretation	2.50(0.70)	2.16(0.83)	1.98	0.50	0.46
Modified Hayling Test	4.84(1.62)	2.36(1.86)	6.46	<0.001	1.42
Total	21.05(4.11)	15.6(4.28)	5.83	<0.001	1.32

* Subtests shared by both IFS and FAB tests. IFS, INECO Frontal Screening; FAB: Frontal Assessment Battery; HC: healthy controls; MCI: mild cognitive impairment patients; SD: standard deviation; M: mean; d: effect size.

### Sensitivity and specificity of Frontal Assessment Battery and INECO Frontal Screening

**Figure 1:**
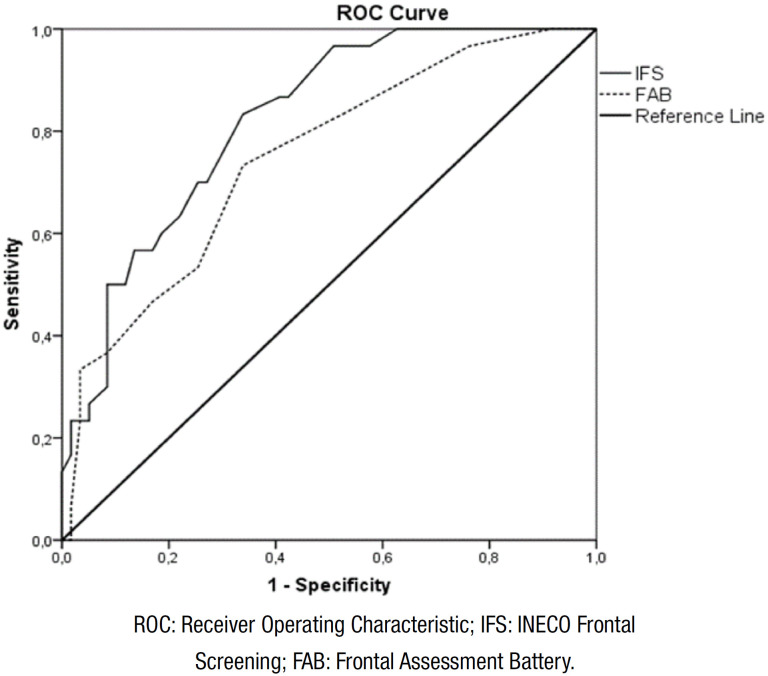
Receiver Operating Characteristic curves show INECO Frontal Screening (total score) and Frontal Assessment Battery (total score) for distinguishing between healthy controls and mild cognitive impairment patients.

Figure 1 shows ROC curves of IFS (total score) and FAB (total score) for detecting MCI (multiple-domain aMCI subtype). The results showed that the area under the curve (AUC) of IFS for MCI patients was .82 (cutoff=20/21; sensitivity=0.90; specificity=0.76), whereas the AUC for FAB was 0.74 (cutoff=13/14; sensitivity=0.96; specificity=0.70) (Table 4).

**Table 4:** Sensitivity, specificity, area under the curve, and cutoff of Frontal Assessment Battery and INECO Frontal Screening for mild cognitive impairment patients vs. healthy controls.

	AUC	95%CI	Cutoff	Sensitivity	Specificity
MCI vs. HC					
IFS	0.82	0.73–0.90	20/21	0.96	0.76
FAB	0.74	0.64–0.85	13/14	0.90	0.70

IFS: INECO Frontal Screening; FAB: Frontal Assessment Battery; MCI: mild cognitive impairment patients; HC: healthy controls; AUC: area under the curve; 95%CI: confidence interval.

## DISCUSSION

The objective of the present study was to compare the sensitivity and specificity of FAB and IFS in patients with amnestic MCI multiple-domain subtype (md-aMCI). First, it was found that years of education showed a positive effect on FAB and IFS, whereas the age of participants did not show a significant effect.

The absence of the effect of age on the IFS score has been previously reported,^18^ ^,^ ^27^ although other authors have found opposite results.^16^ Thus, it is worth developing more studies aimed at verifying the effect of age on IFS scores. The present results did not show an effect of age on the total FAB score, which does not correspond to the results reported in other studies that suggest an inverse effect of age on FAB performance.^28^ ^–^ ^30^

On the other hand, a positive effect of years of education on the global and dimensional scores of FAB and IFS was verified. This result is related to some studies reporting that a higher education level has a positive influence on the performance of various tests that evaluate executive functioning.^31^ ^–^ ^33^ In the particular case of IFS, previous research showed significant effects of education and no significant effects for age on the scores of this instrument.^27^ In another study carried out with patients with dementia, compared with healthy controls, age did not show associations with IFS scores, but with years of education.^18^

The present findings also illustrate that IFS shows better sensitivity than the FAB for distinguishing between healthy controls and MCI patients. In this study, IFS showed higher precision compared with FAB to discriminate between MCI patients and healthy participants. In a recent study, the usefulness of IFS to discriminate between healthy controls and MCI patients was also verified.^16^ The cutoff point reported by the authors is very similar to that found in our study (cutoff =20; sensitivity=0.92; specificity=0.81).

In the specific case of Cuba, we did not find any previous study that explored the clinical utility of FAB; conversely, to date, only one study in the country has investigated the sensitivity and specificity of IFS in detecting cognitive deficits in MCI patients (md-aMCI).^17^ Contrary to results of the present study, the previous study showed that IFS had a low capacity for discriminating between md-aMCI patients and healthy controls. This discrepancy accounts for differences in the cognitive profiles of the MCI patients included in both studies. In the study conducted by Broche-Pérez et al.^17^ the md-aMCI patients did not differ from healthy controls in the following subtests: Conflicting instructions (sensitivity to interference), Months backward, and Digit Span Task (working memory). In this sense, the md-aMCI patients of the present study show a greater executive deficit, which increases the sensitivity of IFS.

It is worth noting that although the existence of executive dysfunctions in MCI patients has been previously published,^34^ the evidence for the utility of brief screening neuropsychological instruments to evaluate them is limited.^16^

In spite of this limitation, the results of this study are related to other research carried out on different neurodegenerative diseases, according to which a greater discriminative capacity of IFS is evidenced compared with FAB. For example, when compared with FAB, IFS has shown greater discriminatory capacity in frontotemporal dementia,^19^ AD, and behavioral variant frontotemporal dementia.^20^ Probably, the superior psychometric properties of IFS in comparison with FAB in the evaluation of MCI patients (md-aMCI subtype) results from the addition of subtest that had demonstrated a high sensitivity to detect subtle executive dysfunctions.^19^

The study had some limitations. First, the MCI group is rather small. In future studies, large samples are needed to confirm statistically significant differences between diagnostic instruments. Furthermore, future studies must delve into the effect of education on performance in FAB and IFS. It is crucial to obtain normative data on the Cuban population according to different years of education in order to prevent biased interpretations and to avoid false-positive or false-negative cases. Additionally, future research should also explore the relationship between results of cognitive screening tests and the structural and functional aspects of brain activity.

In conclusion, the present findings showed that, in comparison with FAB, the IFS presented higher sensitivity for the detection of MCI patients (md-aMCI subtype) with executive dysfunctions. We recommend the inclusion of such test in screening protocols for dementia for the early detection of executive dysfunctions in MCI patients, in all levels of the Cuban Public Health System. The use of IFS in everyday clinical practice would allow detecting frontal dysfunctions in MCI patients with greater precision, facilitating early intervention and impeding the transition to more severe cognitive dysfunctions.
